# Venn Diagrams May Indicate Erroneous Statistical Reasoning in Transcriptomics

**DOI:** 10.3389/fgene.2022.818683

**Published:** 2022-04-14

**Authors:** January Weiner, Benedikt Obermayer, Dieter Beule

**Affiliations:** Core Unit Bioinformatics, Berlin Institute of Health at Charité—Universitätsmedizin Berlin, Berlin, Germany

**Keywords:** gene set analysis, functional genomics, gene set enrichment, transcriptomics, venn diagram

## Abstract

A common application of differential expression analysis is finding genes that are differentially expressed upon treatment in only one out of several groups of samples. One of the approaches is to test for significant difference in expression between treatment and control separately in the two groups, and then select genes that show statistical significance in one group only. This approach is then often combined with a gene set enrichment analysis to find pathways and gene sets regulated by treatment in only this group. Here we show that this procedure is statistically incorrect and that the interaction between treatment and group should be tested instead. Moreover, we show that gene set enrichment analysis applied to such incorrectly defined genes group-specific genes may result in misleading artifacts. Due to the presence of false negatives, genes significant in one, but not the other group are enriched in gene sets which correspond to the overall effect of the treatment. Thus, the results appear related to the problem at hand, but do not reflect the group-specific effect of a treatment. A literature search revealed that more than a quarter of papers which used a Venn diagram to illustrate the results of separate differential analysis have also applied this incorrect reasoning.

## Introduction

Experimental designs for transcriptomic analyses frequently include more than one factor. Often, the question asked is whether there is a difference between groups (first factor) with respect to reaction to a particular treatment (second factor). For example, we may ask whether there are differentially expressed genes (DEGs) which are specific to a particular group of patients, e.g., interferon response elicited by a virus in one group, but absent in another group of patients. In other words, we ask whether the difference between the control group (healthy subjects) and the treatment group (infected patients) is different between two groups of individuals. This “difference of differences” is known in statistics as an interaction (Blalock, 1965). To find out whether it is statistically significant, an appropriate statistical test for interaction should be employed.

However, another approach is widely spread (Nieuwenhuis, Forstmann, and Wagenmakers 2011). Instead of testing the interaction, the effect of the treatment is tested separately in both groups. Next, a difference between groups is inferred if the effect of treatment is significant in one comparison, but not significant in the other. This approach is not correct from statistical point of view, as “the difference between significant and not significant is not itself statistically significant” (Gelman and Stern 2006). For example, the *p*-value in the first comparison may be 0.009, and in the other comparison 0.011. At an alpha level of 0.01 the difference will be statistically significant in the first, but not significant in the other comparison.

**FIGURE 1:**
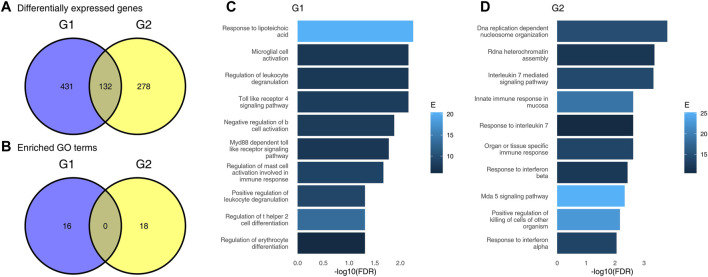
Results of differential gene expression analysis and gene set enrichment analysis using an incorrect approach. **(A)**, Venn diagram showing numbers of differentially expressed genes (DEG) in each of the two groups, G1 and G2; **(B)**, Venn diagram showing numbers of significantly enriched GO terms in each of the two groups; **(C)** results of gene set enrichment analysis for genes “specific” to group G1; **(D)**, results of gene set enrichment analysis for genes “specific” to group G2 (only top 10 terms are shown).

In transcriptomics, statistical tests are performed for thousands of genes. As in the general case, the inference of differences between the groups should correctly be done by testing the significance of interaction between the group and the treatment. In practice, the differences between treatment and control are frequently tested in the two groups separately. This can be visualized using a Venn diagram (VD, Figure 1) showing the overall number of DEGs significant in both comparisons (the intersection in the VD) or significant in only one comparison (the remaining two fields on a VD). The genes which are significant in only one comparison are sometimes incorrectly considered as specific for the corresponding group. Following this, gene set enrichment analysis may be used in an attempt to test which pathways are specific to one, but not the other group.

In this paper, we show that under reasonable assumptions this approach may result in apparent enrichments even if there are no real statistically significant differences between the groups. To this end, we randomly split a cohort into two groups, compared the treatment (viral infection) with controls in each of the groups separately and then applied gene set enrichment to the sets of genes significantly different in one, but not the other group. Moreover, we show that the resulting gene set enrichments correspond to the differential expression between treatment and control. Thus, the enriched terms are relevant to the biological question at hand, yet while they do reflect real processes linked to viral infection, they do not correspond to the differences between the study groups. Finally, we use literature search to show that this incorrect approach to study group-specific treatment effects is not uncommon. In fact, while VDs are a useful visualization tool also in transcriptomics, in more than quarter of the papers where VDs were used, group-specific genes were defined as significant in one, but not other groups, and in 19% of the papers a gene set enrichment was performed.

## Results

### Transcriptomic Changes due to Sars-Cov-2 Infection

Consider two group of patients, G1 and G2 (Table 1). Each group contains 40 individuals. In both groups, there is an equal number of healthy individuals (labeled “Ctrl” on figures below) or patients infected with Sars-Cov-2 (labeled “SC2”). Our aim is to understand the differences between G1 and G2 in the response to infection. For example, we ask which genes or pathways are specifically upregulated by SC2 infection in G1 as compared to G2, and vice versa. In the following, we used the data set GSE156063 (Mick et al., 2020) in two approaches (an incorrect and the correct one) to arrive at opposite conclusions.

**TABLE 1:** Overall design in the case study: transcriptomic changes due to Sars-Cov-2 infection. The table shows number of patients in each combination of study group/disease status.

		Study Group	
		Group 1 (G1)	Group 2 (G2)
Disease status	Sars-Cov-2 infection	20	20
	Another infection	20	20

First, we have performed differential gene expression analysis for each of the groups G1 and G2 separately using standard bioinformatic tools. For each comparison, we defined DEGs as genes for which the false discovery rate (FDR) was lower than 0.05 and absolute log_2_ fold change (LFC) was higher than 1. There were 563 DEGs in the G1 group, and 410 in the G2 group. In total, 132 DEGs were common for G1 and G2, 431 DEGs were significant in G1 only (“specific” for G1), and 278 were significant in G2 only (see Figure 1A). A naive interpretation of these results implies that there is a substantial difference between these two groups of individuals, as evidenced by a small overlap in commonly regulated genes. The majority of DEGs is significant in one comparison only.

To understand which pathways are upregulated in each of the two groups, we used a standard generation I gene set enrichment analysis—a hypergeometric test—on the DEGs in each group. Gene sets for the gene set enrichment analysis were taken from the Gene Ontology (GO) database. Gene sets with more than 50 or fewer than 10 genes were removed. For each group, we have selected only genes which are DEGs in that group, but not the other, mimicking a naive approach for finding pathways regulated in one patient group only. Here, a similar picture emerged. Overall, 16 gene sets were significantly enriched in G1, and 18 gene sets were significantly enriched in G2. Both the Venn diagram (Figure 1B) and the results of enrichments (Figures 1C,D) suggest that there is a fundamental difference between the groups, and that the groups have little in common in their response to the virus.

Importantly, the different GO terms enriched in the two groups were related to infection, and may tempt to speculate about the underlying biological differences between these two groups. For example, the significance of Toll like receptor 4 pathway in G1, but not G2; and, vice versa, significance of response to interleukin 7 in G2, but not in G1 may be considered as evidence of altered immune response to the virus in G2 as compared to G1.

**FIGURE 2:**
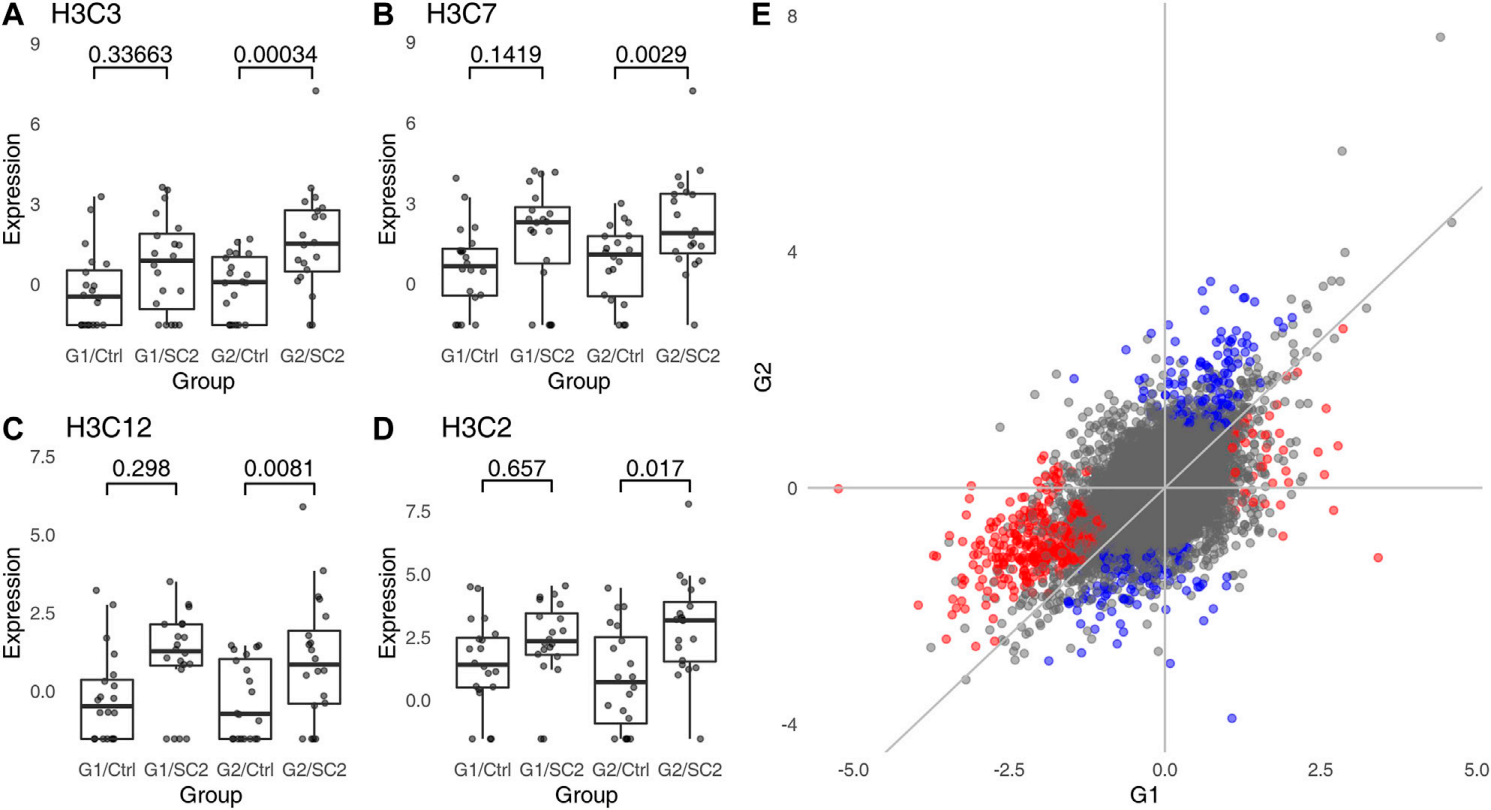
Genes which are significant in one comparison, but not the other do not show a statistically significant interaction. **(A–D)**, examples of genes which are DEG in one group, but are not significantly different in the other group. “Ctrl,” healthy individuals; “SC2,” Sars-Cov-2 infected patients. Values above the plot indicate FDR (*p*-values corrected for multiple testing). **(E)**, correlation between log_2_ fold changes in G1 and G2. Color indicates genes which are significant in one, but not significant in the other comparison; red indicates genes significant in G1, blue indicates genes significant in G2. The overall Pearson correlation coefficient between log_2_ fold changes is 0.55.

However, the groups G1 and G2 were randomly sampled from the same data set. In fact, repeated re-sampling always results in some genes being found to be significantly different in one group, but not the other, despite the fact that one does not expect any major differences between sets of individuals randomly drawn from a single population. Thus, the conclusions drawn from a Venn diagram-driven gene set enrichment analysis are based on artifacts. Closer inspection of genes which are DEGs in one group, but not the other reveals the underlying statistical fallacy (Figures 2A–D), that is, that difference between significant and non-significant is, in itself, not statistically significant (Gelman and Stern 2006). This does not necessarily mean that there are no differences at all between these two groups, but that lack of significance in one group and significance in the other group does not correctly identify differences between groups.

To find genes which are differentially regulated in the two groups, the correct statistical approach is to calculate interaction between groups (G1, G2) and disease status (no disease vs. COVID). While it may be argued that a test for interaction has lower power than a test for a simple contrast, no genes show a significant interaction even at FDR <0.1. In fact, this is not surprising. The log_2_ fold changes for comparisons withing G1 and G2 are strongly correlated (Figure 2E). For all significant genes, the Pearson correlation coefficient is 0.72, while for genes exclusively significant in G1 or G2 (genes “specific” to G1 or G2), it is 0.7 and 0.73, respectively. Thus, genes which are significant in one, but not in the other comparison tend to have similar log_2_ fold changes in both groups (e.g., Figures 2A,C,D).

Consequently, it is not possible to calculate gene set enrichment for the interaction using a hypergeometric test, as there are no DEGs for the interaction contrast. Gene set enrichment using a second generation algorithm (CERNO), relying on the ordering of genes according to their raw *p*-values from the interaction contrast rather than selecting a set of DEGs (Zyla et al., 2019), does not show any significant enrichment.

### Artifacts Arise Because of False Negatives

It is worth noting that in the gene set enrichment analysis of the genes “specific” for a given comparison—i.e., genes which are significant in that comparison, but not significant in others—we have observed a number of terms associated with immune response. It is a crucial point of this manuscript to note that the spurious enrichments not only show significant *p*-values, but also that the terms or pathways which appear in them are relevant to the research hypothesis being tested. Below, we will show why these terms (rather than random terms which have no obvious relevance to an infectious disease) appear in the results.

To understand how significant results appear in a gene set enrichment analysis in randomly generated groups despite absence of genes with significant interaction, it is first necessary to consider the definition of a differentially expressed gene in this context. More often than not, DEGs are defined by a threshold in *p*-value adjusted for multiple testing, possibly combined with a threshold in log_2_ fold change. The commonly used Benjamini-Hochberg procedure (Benjamini and Hochberg 1995) ensures that among genes for which FDR <0.05 there are at most 5% false positives irrespective of the sample size.

This way, we can exert control over the false positive rate (FPR, type I errors), keeping it at a relatively low level. However, we do not control the false negative rate (FNR, type II errors). In a powerful statistical test (such as a t-test), the test power in a typical application will rarely achieve more than 80%. For example, even for large effects (Cohen’s d > 0.8) and type I error rate of 0.05, a t-test only achieves 80% power with at least 25 samples per group. For small effects (Cohen’s d > 0.2), the required number of samples is at least 393 per group. Even assuming a test power of 80%, the FNR is 20%. Clearly, false negatives (FNs) occur at much higher rates than false positives (FPs). In the case of high throughput data sets, where the FPR is controlled by Bejnamini-Hochberg procedure or a similar technique, the FNR may be even as high as 80% (White, Ende, and Nichols 2019).

**FIGURE 3:**
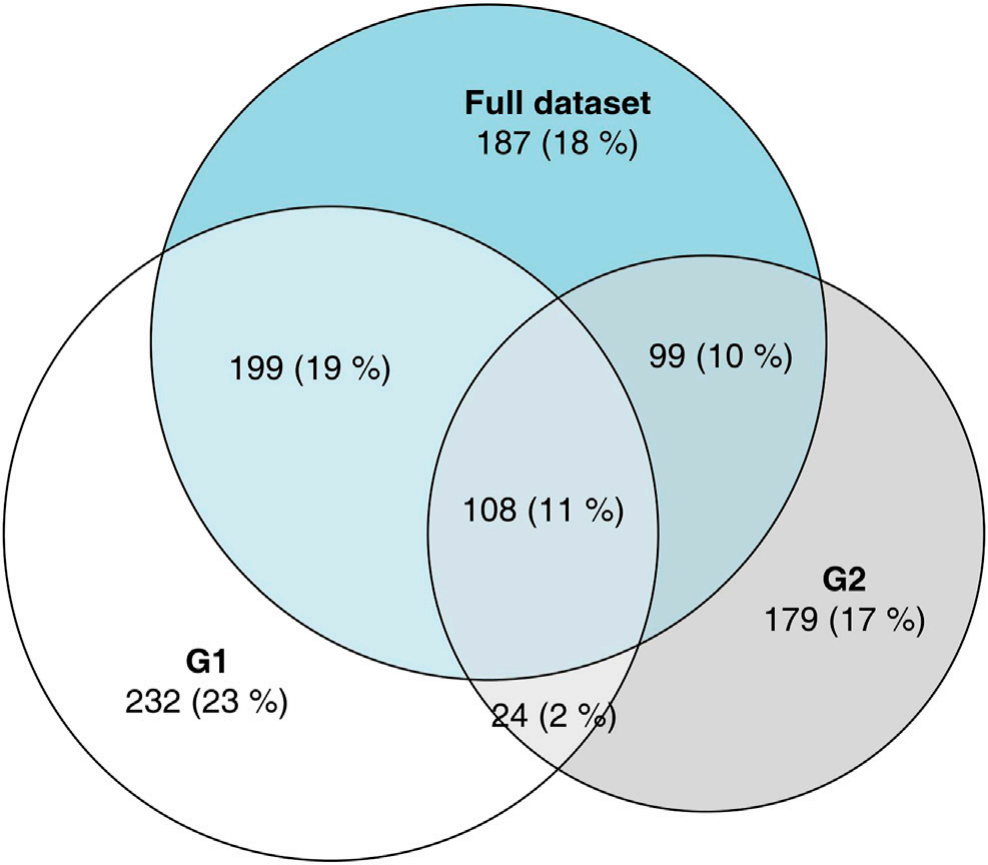
Area-proportional Venn diagram showing overlaps in DEGs between G1, G2 and the full data set. The majority of genes which have been labeled as DEGs in only one of the groups G1, G2 are DEGs when all data were analyzed.

These FNs occur at a much higher rate within the sets of DEGs defined by the non-overlapping areas of the VDs, that is DEGs considered to be “specific” for one group or other in a naive approach. To illustrate this phenomenon, we have analyzed the full data set from which G1 and G2 were drawn (Figure 3), comparing the 100 healthy controls to 93 COVID-19 patients. Of the 431 genes significant in G1, but not in G2, 199 (46%) are significant in the full data set; of the 278 genes significant in G2, but not in G1, 99 (36%) are significant in the full data set. Given that G1 and G2 were sampled from the total population, and since the FDR was set to 0.05, we do not expect more than 30 FPs in the full data set, which implicates that at least 268 out of the 709 “specific” DEGs are true positives in the full data set. Thus, we can assume that at least a third of the genes that appeared to be “specific” in the initial analysis were, in fact, false negatives in one of the comparisons.

In other words, a substantial fraction of the “specific” genes are genes that are in reality differentially expressed in both groups alike. Therefore, if one is to perform a gene set enrichment analysis on one of these “specific” groups of genes, then the enriched functions will be related to the pathways and processes up- or downregulated in both groups due to the common factor (in this example, the COVID-19 disease), but which are not related to differences between the two groups.

### Influence of Sample Size and Cut-Off Thresholds on Number of Artifacts

In the example above, the groups have been randomly sampled from a larger data set only once. Arguably, the observations might differ if the groups were to be resampled. Furthermore, we have chosen a group size of 40 (20 per group/treatment combination). Larger sample sizes are known to increase robustness of gene set enrichment analysis, and group size of 20 has been shown to be relatively robust (Maleki et al., 2019). However, a smaller or larger group size might change the proportion of FNs in the results and thus influence the results of gene set enrichment. Finally, we have used a log_2_ fold change threshold of 1, because raising it would increase the fraction of FNs. However, filtering for biologically relevant genes with a substantially higher effect size may influence the observed enrichments. On the other hand, raising the log_2_ fold change threshold or lowering the sample size may lead to a smaller number of defined DEGs, thus making the hypergeometric test less powerful and lead to fewer gene set enrichment.

To investigate whether the sampling had an impact on the artifacts, we have repeated the above procedure for 100 replicates, each containing a different set of samples randomly assigned to the two groups. Furthermore, we tested whether the selection of the log_2_ fold change threshold or different sample size might have an impact on the extent of the arising artifacts. To this end, we have tested 7 different threshold values for the log_2_ fold changes and three sample sizes: 20, 40 or 80 samples per group (corresponding to 10, 20 or 40 samples per group/treatment combination).

**FIGURE 4:**
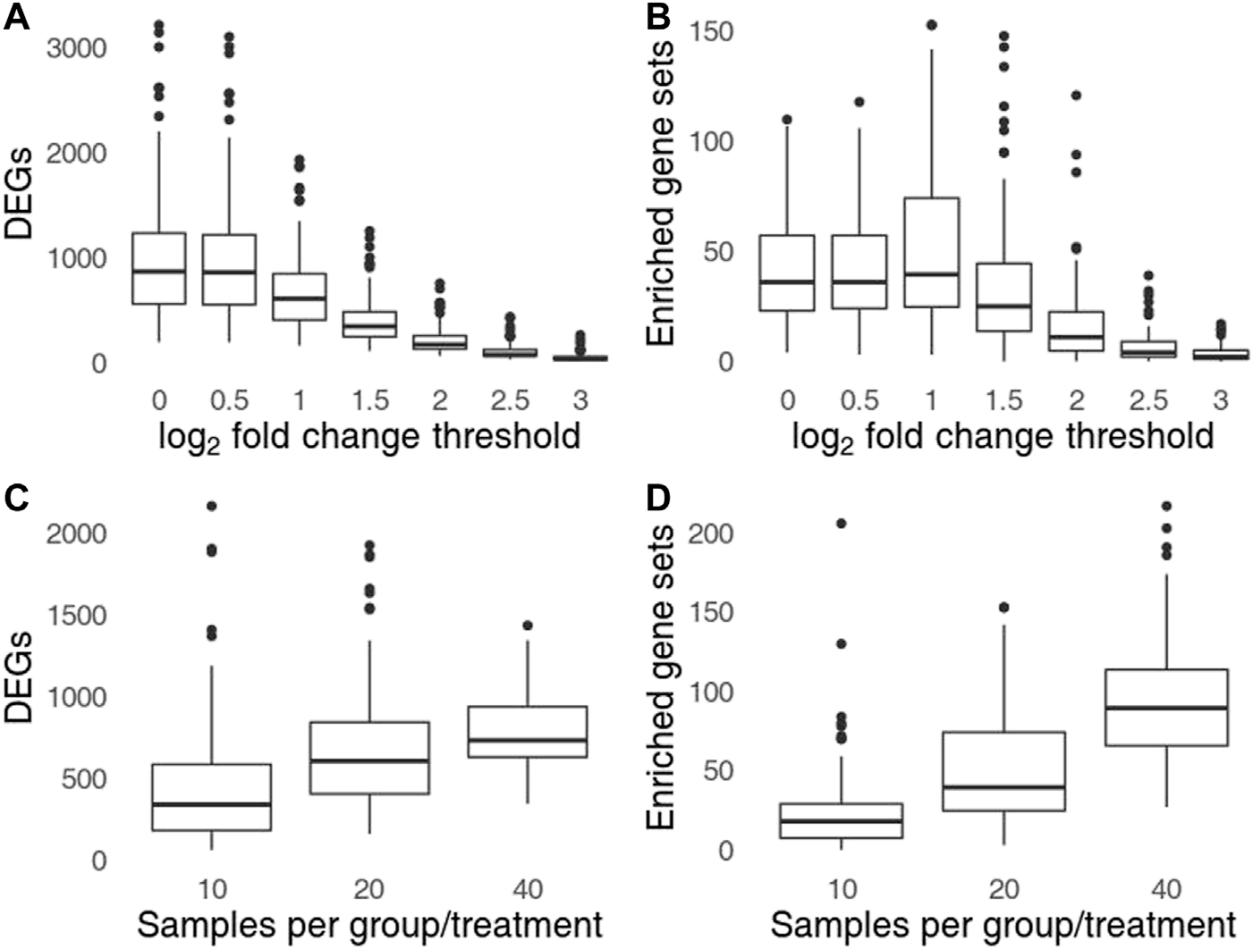
Influence of log_2_ fold change treshold **(A,C)** and the sample size **(B,D)** on the number of genes significant in one, but not in the other group **(A,B)** and the number of significant gene sets found in one group, but not the other **(C,D)**. Top row shows the influence of log_2_ fold change threshold for sample size of 40 per group (20 per group/treatment combination), bottom row shows the influence of sample size for a log_2_ fold change threshold set to 1.

Setting a higher log_2_ fold change threshold reduces the number of DEGs as well as of the observed artifactual gene set enrichments (Figures 4A,B). However, even for log_2_ threshold of 3 (DEGs defined by 8-fold change and FDR <0.05) the number of replicates in which artifacts can be observed is 78 (out of 100), and in at least 31 replicates, 5 or more gene sets were significantly enriched. Thus, setting a more conservative threshold while retaining the incorrect procedure cannot fully protect from the arising artifacts. The number of artifacts rose with sample size (Figures 4C,D). For total sample size of 160 (40 samples per group/treatment combination) the number of artifacts was almost 2 times higher than for total sample size of 80.

### Incorrect Analysis of Interactions is Common in Transcriptomics

Nieuwenhuis, Forstmann, and Wagenmakers (2011) observed incorrect analyses of interactions in about half of the analyzed papers from top neuroscience journals where the authors considered an experimental design allowing for a test for interaction. We wanted to know if this problem is common in transcriptomics, too. To this end, we have searched three journals—from broad to specialized—for the occurrence of the terms “differential expression” with “venn diagrams” (Table 2). Next, we analyzed the selected papers from year 2020 (and years 2015–2020 in one case) to decide whether the VD was described or referred to as showing genes “specific” or “unique” to a particular group or whether a test for interaction was performed. Finally, we checked whether gene set enrichment analysis was applied to genes significant in one, but not the other group in order to find group-specific differences.

**TABLE 2:** Results of the informal literature survey. We searched for papers using Google Scholar and the keywords “venn diagram” and “differential expression.” Journal, journal title; Years, publication dates; Total, total number of papers found using the search phrase; Analyzed, total number of papers which have been analyzed for correctness; Incorrect, total number (percentage) of papers in which the Venn diagram was combined with comparing significance to non-significance; Enrichment, total number (percentage) of papers which combined the Venn diagram with a gene set enrichment analysis.

Journal	Years	Total	Analyzed	Incorrect	Enrichment
Nature Communications	2020	127	30	9 (30%)	6 (20%)
Science Immunology	2015–2020	14	14	6 (43%)	5 (36%)
Scientific Reports	2020	238	238	73 (31%)	42 (18%)
Total		379	282	88 (31%)	53 (19%)

We found that of the 282 analyzed articles which used the terms “venn diagram” and “differential expression,” at least 88 (31%) were using Venn diagrams to compare statistical significance with lack thereof by referring to “unique,” “specific,” “solely regulated” or “exclusive” DEGs. Out of these, at least 53 coupled the VDs with some form of gene set enrichment analysis on the set of supposedly “specific” DEGs. In summary, in at least a quarter of the papers on differential expression in which a Venn diagram was used, it was illustrating an incorrect statistical procedure which may result in artifactual gene set enrichments.

## Discussion

Drawing conclusions from comparing significance with lack thereof is a common statistical fallacy (Gelman and Stern 2006). Just as absence of evidence is not evidence of absence, the failure to reject the null hypothesis does not constitute the same level of evidence as rejecting it. However, when such an incorrect analysis is combined with downstream functional analysis, the resulting pathways or gene ontologies are misleadingly relevant. For example, the identified gene sets are associated with immune response for a research hypotheses involving an infectious disease, or cancer pathways if the underlying research hypothesis involved cancer treatment. Such results may appear reasonable in the given context, especially if the correct analysis of interactions does not show any significant differences. This effect is persistent or even exacerbated for larger sample sizes (Figure 4).

We found that this type of incorrect analysis occurs in more than a quarter of papers where the procedure was illustrated with a VD. That is not to say that VDs are not a useful tool, even in the context of transcriptomics and gene set enrichments, if used correctly. For example, gene set enrichment analysis of an intersection of DEGs (i.e., by considering genes from the overlap in a VD) is not an incorrect procedure. Genes in the overlapping part of a VD are significant in both (or all) comparisons, hence no comparison between significance and non-significance is made.

While VDs appear to be frequently associated with an incorrect statistical reasoning, the use of VDs is not the cause. In the absence of a VD illustrating the DEGs common and unique to the different study groups, two incorrect approaches may still be found. Firstly, the direct comparison of gene set enrichment results: that is, drawing conclusions from the fact that a gene set enrichment result was significant in one comparison only. Second, while VDs are often used to illustrate the numbers of “specific” DEGs and so present a mean to find examples of this fallacy in scientific literature, researches test for enrichment these “specific” genes without using the phrase “Venn diagram” or even clearly stating how the lists of “specific” genes were derived. In all these cases, the analysis boils down to comparing results significant in one, but not in another comparison.

As an alternative to Venn diagrams and the downstream gene set enrichment analysis, two approaches can be considered. The correct statistical approach, as shown above, involves a test for interaction which can reveal genes for which the impact of treatment significantly differ between the groups. The results can then be plugged into a gene set enrichment analysis the usual way. Unfortunately, this has two major drawbacks. Firstly, effect sizes (log_2_ fold changes) of the interaction term are harder to interpret than log_2_ fold changes in a direct, group vs. group comparison. The effect size in an interaction is negative if the log_2_ fold change in the first comparison is larger than the log_2_ fold change in the second comparison; this is, however, irrespective of whether the differences in the individual comparisons are negative or positive, which makes it harder to separate the differences in genes upregulated in one or both groups.

The second problem may arise if the changes are similar in both comparisons, but of larger magnitude in one of them. For example, in a time series context, the changes may be more pronounced at a later time point. In this case, the analysis will show that the processes enriched for the interaction term are the same as those enriched in each of the comparisons individually. While the results of the gene set enrichment analysis in this context are correct, the result may not be what the researchers intended—processes which qualitatively (rather than quantitatively) differ between the comparisons.

An alternative approach, discordance/concordance analysis, has been proposed by Domaszewska et al. (2017), aiming at identifying processes which qualitatively differ between the two comparisons. Here, a heuristic score (“disco score”) has been defined which depends on the effect sizes and *p*-values in both comparisons. The sign of the score depends on whether the effects have the same sign (concordant; genes upregulated in both comparisons or downregulated in both comparisons) or opposite signs (discordant; genes upregulated in one, but downregulated in the other comparison, and genes downregulated in one, but upregulated in the other comparison). While the score does not allow the calculation of a *p*-value and does not present an alternative to an analysis of interaction, it can facilitate both visualization and further analysis using a gene set enrichment algorithm.

Visualization of interaction for individual genes is straightforward (see for example Figures 2A–D). However, the point of VDs is to show a grand overview of the whole analysis summarizing thousands of results for the analyzed genes. As an alternative of such an overview, we suggest plotting the log∼2 fold changes in one comparison against log_2_ fold changes in the second comparison. This allows an intuitive assessment of the differences between the two comparisons, in especially in combination with color coding the genes which either are significant in the interaction or by coloring using the disco score (see Figure 2E for an example).

Defining group-specific genes based on significant difference in one, but no significant difference in another comparison is thus more than only a statistical fallacy leading to erroneous results. When combined with gene set enrichment analysis it can lead to potentially sound-looking, and therefore particularly misleading results. This method of obtaining specific differences between groups should therefore be abandoned in favor of statistically correct approaches. Furthermore, gene set enrichment analysis must never be applied to sets of genes defined as significant in one comparison, but not the other.

## Methods

### Methods Availability

This manuscript has been written in R markdown (Xie, Allaire, and Grolemund 2018).All statistical calculations required to replicate the findings and figures are contained in the source R markdown file. The R markdown file, along with additional files required to recreate this manuscript as well as the results of literature survey have been uploaded to https://github.com/bihealth/manuscript_venn_diagrams.

### Data

The expression data as a count matrix has been downloaded from GEO, accession GSE156063.

### Statistical Analyses

Power calculation was done using the R package pwr, version 1.3.0. For differential gene expression, the R package DESeq2, version 1.32.0 has been used. Gene set enrichments were done using either hypergeometric test (where stated) or the CERNO test using the package tmod (Zyla et al., 2019), version 0.50.1. GO terms have been sourced from the R package msigdbr, version 7.4.1.

### Simulation Study

We have generated replicates of the example study for different log_2_ fold change thresholds (0, 0.5, 1, 1.5, 2, 2.5, 3) and three different sample sizes (40, 80 and 160, corresponding to sample size per group/treatment combination of 10, 20 and 40). For each replicate, the full procedure as described above was repeated, and numbers of DEGs and significantly enriched terms were collected.

### Literature Survey

A literature survey was performed using Google Scholar to estimate the frequency of the incorrect use of Venn diagrams. We searched for articles including the phrases “differential expression” and “venn diagram” in three journals: Scientific Reports (2020), Nature Communications (2020) and Science Immunology (2015–2020). For each of the papers identified, we checked whether 1) the authors used the VD to show differentially expressed transcripts significant in one comparison, but not another, 2) the authors discussed “unique,” “non-overlapping” or “specific” regions of the Venn diagram and 3) whether this was coupled to gene set enrichment analysis is any form. Articles which 1) focused only on the intersections of the Venn diagrams (genes common to all groups), or 2) which used the Venn diagrams for a purpose other than to compare genes significant in one groups, but not significant in other groups or 3) for which a clear-cut error could not be identified past any reasonable doubt were not considered incorrect. The results of the literature survey (including links to papers classified as incorrectly analysing the interaction) are included in the manuscript sources.

## Data Availability

Publicly available datasets were analyzed in this study. This data can be found here: https://www.ncbi.nlm.nih.gov/geo/query/acc.cgi?acc=GSE156063.

## References

[B1] Benjamini Y. Hochberg Y. (1995). Controlling the False Discovery Rate: A Practical and Powerful Approach to Multiple Testing. J. R. Stat. Soc. Ser. B (Methodological) 57 (1), 289–300. 10.1111/j.2517-6161.1995.tb02031.x

[B2] Blalock H. M. Jr (1965). Theory Building and the Statistical Concept of Interaction. Am. Sociological Rev. 30, 374–380. 10.2307/2090718 14286875

[B3] Domaszewska T. Scheuermann L. Hahnke K. Mollenkopf H. Dorhoi A. Kaufmann S. H. E. (2017). Concordant and Discordant Gene Expression Patterns in Mouse Strains Identify Best-Fit Animal Model for Human Tuberculosis. Sci. Rep. 7 (1), 12094–12113. 10.1038/s41598-017-11812-x 28935874 PMC5608750

[B4] Gelman A. Stern H. (2006). The Difference between "Significant" and "Not Significant" Is Not Itself Statistically Significant. The Am. Statistician 60 (4), 328–331. 10.1198/000313006x152649

[B5] Maleki F. Ovens K. McQuillan I. Kusalik A. J. (2019). Size Matters: How Sample Size Affects the Reproducibility and Specificity of Gene Set Analysis. Hum. Genomics 13 (1), 42–12. 10.1186/s40246-019-0226-2 31639047 PMC6805317

[B6] Mick E. Kamm J. Pisco A. O. Ratnasiri K. BabikBabik J. M. Castañeda G. (2020). Upper Airway Gene Expression Reveals Suppressed Immune Responses to SARS-CoV-2 Compared with Other Respiratory Viruses. Nat. Commun. 11 (1), 5854–5857. 10.1038/s41467-020-19587-y 33203890 PMC7673985

[B7] Nieuwenhuis S. ForstmannForstmann B. U. Wagenmakers E.-J. (2011). Erroneous Analyses of Interactions in Neuroscience: A Problem of Significance. Nat. Neurosci. 14 (9), 1105–1107. 10.1038/nn.2886 21878926

[B8] White T. van der Ende J. Nichols T. E. (2019). Beyond Bonferroni Revisited: Concerns over Inflated False Positive Research Findings in the Fields of Conservation Genetics, Biology, and Medicine. Conserv Genet. 20 (4), 927–937. 10.1007/s10592-019-01178-0

[B9] Xie Y. Joseph J. A. Grolemund G. (2018). R Markdown: The Definitive Guide. Boka Raton, London and New York: Chapman; Hall/CRC.

[B10] Zyla J. Marczyk M. Domaszewska T. Kaufmann S. H. E. Polanska J. Weiner J. 3rd (2019). Gene Set Enrichment for Reproducible Science: Comparison of CERNO and Eight Other Algorithms. Bioinformatics 35 (24), 5146–5154. 10.1093/bioinformatics/btz447 31165139 PMC6954644

